# The preclinical gap in pancreatic cancer and radiotherapy

**DOI:** 10.1242/dmm.050703

**Published:** 2024-07-09

**Authors:** Mathias Tesson, Jennifer P. Morton

**Affiliations:** ^1^ Cancer Research UK Scotland Institute, Switchback Rd, Glasgow G61 1BD, UK; ^2^ School of Cancer Sciences, University of Glasgow, Glasgow G61 1QH, UK

**Keywords:** Pancreatic cancer, Preclinical research, Radiotherapy

## Abstract

Pancreatic ductal adenocarcinoma is an aggressive malignancy with limited treatment options. Chemotherapy offers little benefit and, although there is some evidence that radiotherapy may improve response, its use in the clinical management of pancreatic cancer remains controversial due to conflicting reports on its survival benefit. There has also been a lack of clinical trials that directly investigate the efficacy of radiotherapy in pancreatic cancer. The limited progress in the development of radiotherapeutic strategies in pancreatic cancer can be attributed, at least in part, to a dearth of preclinical research and our limited understanding of the effects of radiation on the pancreatic tumour microenvironment. In this Perspective, we discuss how insight into the immunosuppressive tumour microenvironment and the complex signalling between tumour and stromal cells following radiation is needed to develop effective radiosensitising strategies for pancreatic cancer. We also highlight that to have the best chance for successful clinical translation, more preclinical research is required in appropriately complex models.

## Teaser

**Summary:** Radiotherapy for pancreatic cancer management is the subject of ongoing debate. We discuss how the lack of preclinical work assessing its impact in the tumour microenvironment may be restricting progress.


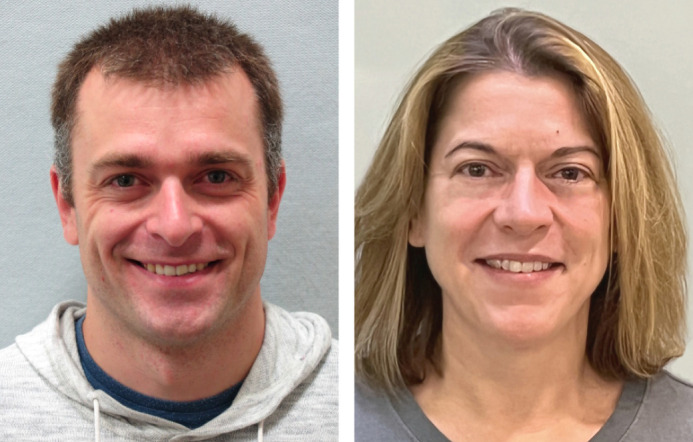


**Mathias Tesson (left) and Jennifer P. Morton (right)**

## Introduction

Pancreatic ductal adenocarcinoma (PDAC) is a devastating disease with a 5-year survival rate of less than 10%. It is predicted to rank as the second leading cause of cancer deaths by 2030, highlighting the need for improved treatment options (Rahib et al., 2021). The only potentially curative treatment is surgery but, in most cases, surgery is precluded by anatomical location or metastatic spread. Current conventional chemotherapeutic approaches are ineffective and, despite promising data in other cancer types, immunotherapy has also proved ineffective in pancreatic cancer (Bockorny et al., 2022). In resectable (operable) or borderline resectable cases, neoadjuvant chemotherapy can increase the likelihood of complete surgical removal of the cancer cells, whereas in locally advanced disease, neoadjuvant therapy may be palliative. However, distinguishing patients with localised disease from those with micro-metastatic disease remains a challenge.

There is some evidence that neoadjuvant chemo-radiotherapy confers better local control of tumours compared with chemotherapy alone (Janssen et al., 2022). However, the use of radiotherapy for patients with pancreatic cancer is still controversial, with several trials reporting conflicting results (Ejlsmark et al., 2023). Chemo-radiotherapy has been shown to delay local tumour progression but with no improvement in overall survival (Hammel et al., 2016). Conversely, the PREOPANC phase III trial (NCT04927780) reported that neoadjuvant radiotherapy with the chemotherapeutic gemcitabine improved overall survival and resection rate in patients with resectable or borderline resectable disease (Versteijne et al., 2020, 2022). The shortage of trials directly assessing the contribution of radiotherapy in the perioperative setting has, however, limited its application in pancreatic cancer. Indeed, few of the ongoing trials including radiotherapy for pancreatic cancer are designed to specifically address the efficacy of radiotherapy (Table 1).

**Table 1.**
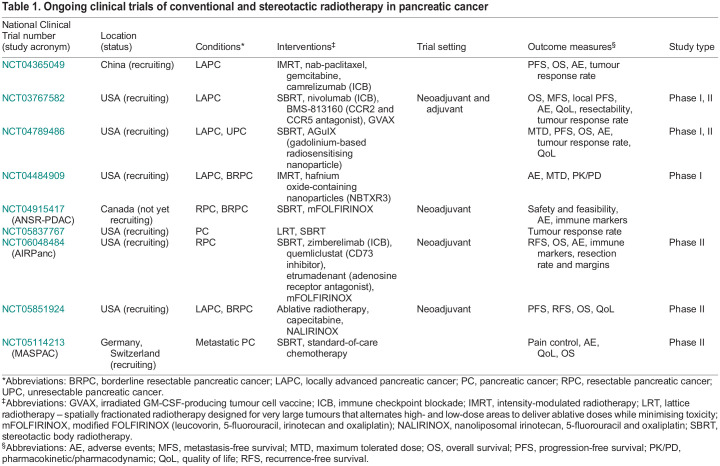
Ongoing clinical trials of conventional and stereotactic radiotherapy in pancreatic cancer

A lack of clinically relevant preclinical research has contributed to a dearth of understanding of the effects of radiation on the pancreatic tumour microenvironment.

The lack of consistent survival benefit conferred by radiotherapy, despite better loco-regional control in some studies, may be due to undetected metastasis at diagnosis, or to both intrinsic and acquired radioresistance. Better pathological outcomes observed in some trials indicate that at least a subset of patients could benefit from neoadjuvant radiotherapy. However, insights into the mechanisms of radiosensitivity or resistance are needed to increase the number of patients who might benefit from radiotherapy, or for patient selection to be possible. Resistance to radiation can occur due to enhanced DNA damage repair, for instance, through activation of ATM kinase, ATR kinase and DNA-dependent protein kinase (DNA-PK), induction of cell cycle arrest, pro-survival signalling, autophagy induction or inhibition of apoptosis. Consequently, tumours harbouring mutations that affect these pathways may have increased radiosensitivity. Hypoxia and metabolic reprogramming of tumour cells also contribute to radioresistance (Li et al., 2012; Brunner et al., 2005; Souchek et al., 2014; Yu et al., 2021). The contribution of the tumour microenvironment (TME) to radioresistance is also likely key. Radiation affects the TME by inducing fibrosis, extracellular matrix remodelling, inflammation and vascular damage, all of which may enable tumour cell survival, dissemination and radioresistance (Krisnawan et al., 2020). Radiation can also result in both tumour-suppressive and tumour-promoting changes in the immune microenvironment, on the one hand, by priming anti-tumour immunity through the release of tumour-derived antigens, but on the other hand, through toxicity to effector immune cells or upregulation of immunosuppressive factors in the microenvironment (Colton et al., 2020). However, a lack of clinically relevant preclinical research has contributed to a dearth of understanding of the effects of radiation on the pancreatic TME (Ahmad et al., 2019). Only by building a clearer understanding of these changes can we develop effective strategies for improving radiotherapy response in pancreatic cancer. It is therefore vital that preclinical research is performed in appropriately complex preclinical models to improve the chances of successful clinical translation.

## Effects of radiotherapy on the PDAC TME

In pancreatic cancer, the fibrotic stroma often constitutes much of the tumour and can have profound effects on tumour progression and therapeutic response. In turn, therapeutic interventions, including radiotherapy, may have significant effects on the stroma. Radiotherapeutic strategies have typically focussed on creating DNA damage in proliferating tumour cells. However, many studies have shown that the impact of radiotherapy *in vivo* extends well beyond the tumour cells. Increased DNA damage in tumour cells results in cell death and the release of neoantigens from the dying tumour cells that can elicit an anti-tumoural cytotoxic T cell (CTL) response (Martinez-Zubiaurre et al., 2018). Indeed, radiation in combination with immunotherapy has been shown to promote CTL activity towards pancreatic tumours (Twyman-Saint Victor et al., 2015), and several clinical trials are currently assessing the efficacy of radiotherapy combined with immune checkpoint blockade (ICB) or therapies to combat immune suppression (Table 1). Radiotherapy also drives vascular changes that can promote the passage of immune cells into affected tissues.

**Fig. 1.**
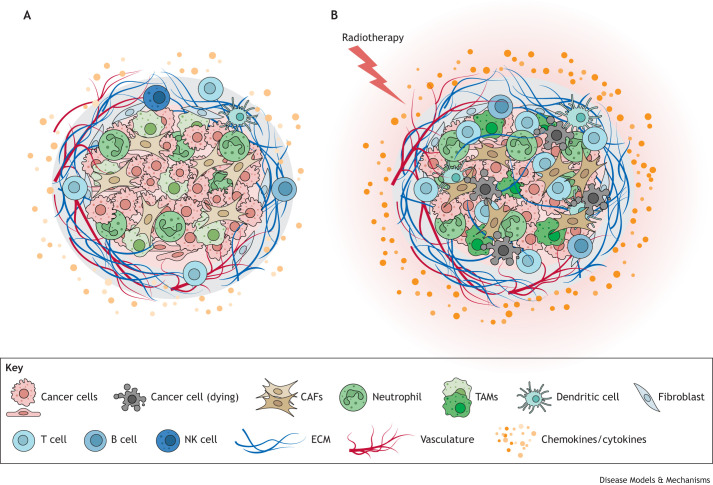
**Effects of radiotherapy on pancreatic cancer.** Pancreatic cancer is characterised by a dense desmoplastic stroma, consisting of cancer-associated fibroblasts (CAFs), extracellular matrix (ECM) components such as collagen, tumour-associated macrophages (TAMs) and neutrophils, but few T cells, B cells or natural killer (NK) cells. These cells are all capable of secreting chemokines and cytokines that can influence other cells. Radiotherapy creates DNA damage, which can result in the death of cancer cells. This may result in the release of neoantigens that can elicit an anti-tumoural T cell response; however, the release of inflammatory signals can also induce alterations in the behaviour of CAFs and macrophages that may favour an immunosuppressive, pro-tumour environment.

In contrast, radiotherapy, and the release of antigens and inflammatory cytokines and chemokines – such as CC-chemokine ligand (CCL) 2 (CCL2), CCL4, CCL5, CXC-chemokine ligand (CXCL) 9 (CXCL9), CXCL10, CXCL12, IFNγ (encoded by *IFNG*), interleukin (IL) 1β (encoded by *IL1B*), IL-4, IL-6, IL-13, iNOS (encoded by *NOS2*), TNFα (encoded by *TNF*) and TGFβ1 (encoded by *TGFB1*) (Ansems and Span, 2020; Cytlak et al., 2022) – can result in tumour-promoting outcomes (Fig. 1). For example, radiotherapy can induce alterations in cancer-associated fibroblast (CAF) secretory behaviour, which can drive fibrosis and extracellular matrix remodelling, as well as tumour-associated macrophage (TAM) expansion and polarisation towards an immunosuppressive phenotype (Cho et al., 2018; Li et al., 2016; Pereira et al., 2020; Hellevik et al., 2021). This macrophage polarisation can, in turn, downregulate the anti-tumour immune response mediated by CTLs and natural killer cells to favour tumour cell survival, metastasis, treatment resistance and maintenance of an immunosuppressive milieu (Krisnawan et al., 2020; Ansems and Span, 2020; Shi and Shiao, 2018; Wirsdorfer and Jendrossek, 2016). Indeed, radiotherapy has been shown to drive fibrosis and an immunosuppressive phenotype in mice bearing pre-neoplastic pancreatic lesions and can accelerate tumour progression (Seifert et al., 2016; Mueller et al., 2021). Furthermore, radiotherapy was shown to significantly alter the immune microenvironment in patients with PDAC, illustrated by changes in the expression and distribution of immune markers in tumours, including lower expression of T cell markers and checkpoint molecules (Farren et al., 2020). Radiation has also been reported to lead to an increase in immunosuppressive TAMs in PDAC models (Jones et al., 2018; Bian et al., 2021). However, although targeting inflammatory mediators could abrogate some of the potentially pro-tumour effects of radiotherapy, it may also constrain DNA damage-mediated anti-tumour immune responses.

Ideally, an effective radiotherapeutic combinatorial strategy would kill proliferating tumour cells while promoting anti-tumourigenic immune responses and inhibiting pro-tumourigenic immune and fibrotic responses.

Ideally, an effective radiotherapeutic combinatorial strategy would kill proliferating tumour cells while promoting anti-tumourigenic immune responses and inhibiting pro-tumourigenic immune and fibrotic responses. However, the mechanisms by which myeloid cell and CAF populations can drive a fibrotic immunosuppressive microenvironment following radiotherapy are poorly understood. Recent studies have highlighted the heterogeneity of immune cells and CAFs in the PDAC TME (Helms et al., 2020; Liudahl et al., 2021), and, to prevent pro-tumourigenic effects of radiotherapy, we most likely need to target specific behaviours. For example, depleting the membrane glycoprotein SIRPα (encoded by *SIRPA*) from macrophages can enhance the efficacy of radiotherapy in subcutaneous tumours resulting from transplant of syngeneic mouse pancreatic cancer cell lines, as SIRPα-deficient macrophages are able to adopt a pro-inflammatory anti-tumour response following irradiation, promoting enhanced CTL and natural killer cell infiltration with limited fibrosis (Bian et al., 2021).

Similarly, although targeting all CAFs was once considered an attractive option, depletion has been shown to promote pancreatic tumourigenesis in certain circumstances (Ozdemir et al., 2015; Rhim et al., 2014); thus, inhibiting specific pro-tumour microenvironmental signalling pathways will be important. For example, CAFs have been shown to limit radiotherapy efficacy by activating signalling through the integrin-associated FAK tyrosine kinase (encoded by *PTK2*), with FAK inhibition rescuing engagement of the adaptive immune response. This study highlighted the discrepancy in radiotherapy response between tumours resulting from intra-pancreas (orthotopic) transplant of syngeneic mouse pancreatic cell lines and pancreatic tumours that spontaneously arise in genetically engineered mouse models (GEMMs) that express the PDAC-promoting mutations *Kras^G12D^* and *Trp53^R172H^* specifically within the pancreas (Lander et al., 2022).

Other efforts to augment the adaptive immune response have achieved some success in terms of reprogramming the immune TME and enhancing response to radiotherapy. This was demonstrated with the administration of IL-12 (Mills et al., 2019) or inhibition of CD73 (encoded by *NT5E*), a surface enzyme involved in the conversion of AMP to the immunosuppressive metabolite adenosine (Ye et al., 2023), in both orthotopic transplant models using syngeneic mouse pancreatic cell lines and spontaneous *Kras^G12D^*- and *Trp53^R172H^*-driven GEMMs of pancreatic cancer. Further strategies include increasing dendritic cell (DC) recruitment and/or priming to drive T cell recruitment and activation, and ICB to overcome T cell inhibition or exhaustion (Twyman-Saint Victor et al., 2015; Hegde et al., 2020; Piper et al., 2022, 2023). Indeed, promoting conventional DC infiltration and activation via FLT3L (encoded by *FLT3LG*), which increases DC proliferation and survival, and CD40 agonism, which supports DC activation, enhanced radiotherapy response (Hegde et al., 2020). Radiotherapy also improved outcomes in response to anti-CD40 in combination with ICB in genetically engineered mice with *Kras^G12D^*- and *Trp53^R172H^*-driven spontaneous pancreatic tumours, and in mice with pancreatic cancer cells derived from the same GEMM transplanted in the pancreas. Again, however, there were significantly more CD8^+^ T cells in transplant tumours than in the GEMM, both before and after treatment (Rech et al., 2018). Clearly, radiotherapy in pancreatic cancer can elicit a range of effects on the behaviour of different cells within the tumour and the crosstalk between them, and preclinical modelling can help shed light on these events.

## Mouse models

**Fig. 2.**
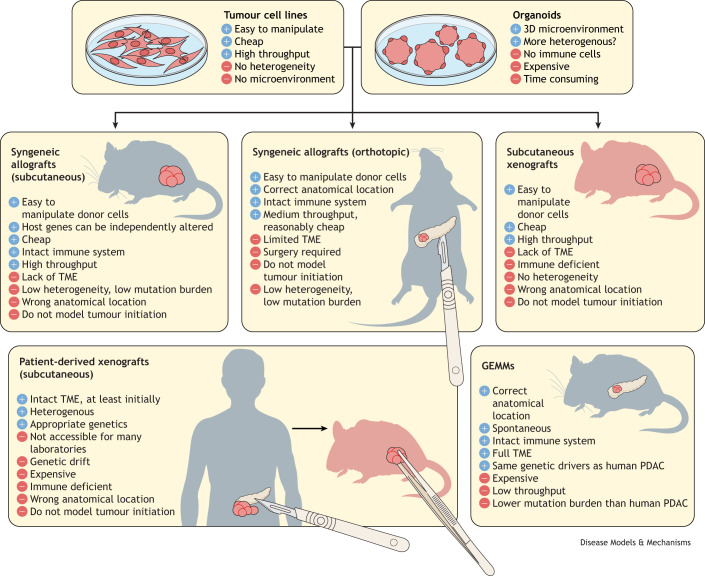
**Advantages and disadvantages of widely used preclinical models of pancreatic cancer.** There are many preclinical models that have been used to study pancreatic cancer, from simple xenografts of human tumour cell lines, xenografts or allografts using tumour organoids, and patient-derived xenografts, to complex genetically engineered mouse models (GEMMs) driven by the same mutations found in human pancreatic ductal adenocarcinoma (PDAC). The advantages and disadvantages of each are highlighted in the figure. 3D, three-dimensional; TME, tumour microenvironment.

Preclinical radiotherapy studies are possible due to the development of precise micro-irradiators that allow targeted radiotherapy in mice, for example, the Small Animal Radiotherapy Research Platform (SARRP) (Wong et al., 2008). A range of preclinical mouse models of pancreatic cancer have been established, ranging from simple xenografts or allografts of human or mouse cancer cell lines, organoids or tumour fragments, to more complex autochthonous GEMMs, in which pancreatic tumours develop spontaneously, driven by the introduction of driver mutations specifically within the pancreas (Vudatha et al., 2023) (Fig. 2). Although murine transplant studies (allografts) can be performed in immunocompetent mice, human tissue transplants (xenografts) require the use of immunodeficient host mice in most cases. The development of immunologically humanised mouse models has enabled xenografting to be performed in the immune competent setting; however, these are prohibitively expensive for many laboratories. As autochthonous models are immunocompetent and the tumours arise spontaneously with co-evolution of the TME, they are likely more predictive, particularly of the efficacy of stromal or immune-targeting therapies. Furthermore, they develop invasive and metastatic tumours over a longer period of time, reflecting the disease state of most patients enrolled in clinical trials. However, due to ease of use, most preclinical radiotherapy studies have been performed in allograft models using syngeneic cell lines or organoids, which lack the complex TME that likely plays a significant role in the response to radiotherapy. Autochthonous mouse models such as the LSL-*Kras^G12D/+^*; LSL-*Trp53^R172H/+^*; *Pdx1-Cre* (KPC) mouse model (Hingorani et al., 2005) and its derivatives develop spontaneous pancreatic cancer that phenocopies the immunosuppressive fibrotic stroma associated with the human disease. Many studies have provided gene expression signatures of radiation effects (Choudhary et al., 2020; Doan et al., 2018; Moreno-Villanueva et al., 2019); however, none have been performed in autochthonous models or considered the differing responses in the constituent cells of the tumour. It seems sensible to investigate radiation responses in the context of a full TME and to view the global effects of radiotherapy to better understand mechanisms of radioresistance and thus identify ways to improve radiosensitivity.

Of course, no one model is perfect in terms of phenocopying the human disease. There are obviously still drawbacks associated with GEMMs. They are unlikely to recapitulate the full genomic diversity of human cancer as tumours develop rapidly in comparison to disease evolution in humans and harbour a lower mutation burden. Furthermore, they do not incorporate the systemic factors that affect tumour progression, for example, ageing, comorbidities or environmental exposures. The field effect – the development of several lesions or tumours within the pancreas – is also an issue in most autochthonous mice. As these models tend to incorporate *Kras* mutations throughout the pancreas, acinar atrophy and inflammation fail to resolve as they would in wild-type tissue, resulting in widespread acinar to ductal metaplasia and lesions throughout the pancreatic parenchyma. This also significantly hampers any opportunity to resect tumours in these models; however, with the advent of mutant Kras inhibitors and the use of optimal radiotherapy regimens, local tumour control could provide a window of opportunity for testing systemic targeted therapies.

The treatment duration for any preclinical study is important, as highlighted by the failure of Hedgehog pathway inhibitors in the clinic following short-term preclinical studies demonstrating promising results (Honkala et al., 2022). Furthermore, as most patients in clinical trials already have advanced disease, models should also reflect these patients, which may not be the case for simpler transplant models. Ideally, we need to benchmark models and therapeutic effects in these models against the human disease as early as possible to make sure the appropriate preclinical models are used and mechanistic studies are relevant.

## Future directions and challenges

Until recently, radiotherapy has been administered as fractionated doses over days or weeks; however, the way in which radiotherapy is delivered is evolving, with intraoperative radiotherapy, hypo-fractionated ablative radiotherapy, high-dose stereotactic body radiation therapy (SBRT) and stereotactic magnetic resonance-guided ablative radiotherapy (SMART), allowing high doses of radiation to be delivered to the tumour in a lower number of fractions while sparing surrounding healthy tissue. Preliminary studies have shown that SBRT may provide local control of pancreatic cancer with minimal toxicity (Ng and Herman, 2018), and further investigation of innovative combinations is ongoing (Benkhaled et al., 2023). Strategies to protect surrounding organs from radiation toxicity, notably the gastrointestinal tract, may also allow ablative radiotherapy to be delivered more safely (Fujimoto et al., 2019; Taniguchi et al., 2023).

Patient selection may also improve outcomes. There are likely tumour cell-intrinsic vulnerabilities that could make chemo-radiotherapy an attractive option in selected patients. DNA damage repair deficiency, for example, through BRCA1/BRCA2, ATM or PALB2 loss, is seen in ∼15% of all pancreatic tumours (Pishvaian et al., 2018) and may represent a window of opportunity for radiotherapy in these patients due to increased radiosensitivity as a result of genomic instability (Karnak et al., 2014). Altered metabolism following radiotherapy may also provide therapeutic opportunities. Indeed, combining radiotherapy with metabolic targets in the TME can improve therapeutic response in some cases (Souchek et al., 2014; Mittal et al., 2022), and interrogating metabolic changes following radiotherapy could reveal novel therapeutic approaches. Many studies have described the metabolic crosstalk in the PDAC TME (Li et al., 2021), but very few, thus far, have examined it in the context of radiotherapy.

There are many challenges remaining but also opportunities. Radiotherapy may provide a window of opportunity for ICB or even cancer vaccines, but, given the complexity of the PDAC TME, the challenge will be to develop effective combinations that limit the deleterious effects of radiotherapy while optimising tumour cell death and immune activation. Even in treatment of naïve pancreatic cancer, knowledge of how different components of the PDAC TME can promote or suppress tumourigenesis and immune evasion is lacking, so understanding the heterogeneity of response to radiotherapy will be important.

Refinement of radiotherapy delivery in the clinic also offers new opportunities, but these should be accompanied by mechanistic studies in the most suitable mouse models.

Refinement of radiotherapy delivery in the clinic also offers new opportunities, but these should be accompanied by mechanistic studies in the most suitable mouse models. The range and complexity of models has grown over the past 20 years, but they need to be carefully validated in terms of fidelity to the human disease and response to therapy. Preclinical models have, at times, been criticised for their lack of predictive value, and this is exacerbated by poor model selection and the lack of visibility for negative preclinical work due to the challenges associated with publishing these studies. We should also consider what constitutes a promising therapeutic strategy preclinically. Short extensions in survival may be statistically significant, but perhaps extended tumour control or regression should be the standard by which to measure success, particularly when many clinical trials are performed in patients with advanced or chemorefractory disease. Improving the predictive power of preclinical modelling and providing confidence in novel preclinical concepts should facilitate translation to the clinic.
